# Surface-enhanced Raman scattering (SERS) revealing chemical variation during biofilm formation: from initial attachment to mature biofilm

**DOI:** 10.1007/s00216-012-6225-y

**Published:** 2012-07-21

**Authors:** Yuanqing Chao, Tong Zhang

**Affiliations:** Environmental Biotechnology Lab, The University of Hong Kong, Pokfulam Road, Hong Kong, SAR China

**Keywords:** SERS, Biofilm formation, Biofilm matrix, Raman microscopy, Atomic force microscopy

## Abstract

Surface-enhanced Raman scattering (SERS) has recently been proved to be a promising technique for characterizing the chemical composition of the biofilm matrix. In the present study, to fully understand the chemical variations during biofilm formation, SERS based on silver colloidal nanoparticles was applied to evaluate the chemical components in the matrix of biofilm at different growth phases, including initial attached bacteria, colonies, and mature biofilm. Meanwhile, atomic force microscopy was also applied to study the changes of biofilm morphology. Three model bacteria, including *Escherichia coli*, *Pseudomonas putida*, and *Bacillus subtilis*, were used to cultivate biofilms. The results showed that the content of carbohydrates, proteins, and nucleic acids in the biofilm matrix increased significantly along with the biofilm growth of the three bacteria judging from the intensities and appearance probabilities of related marker peaks in the SERS spectra. The content of lipids, however, only increased in the Gram-negative biofilms (*E. coli* and *P. putida*) rather than the Gram-positive biofilm (*B. subtilis*). Our findings strongly suggest the SERS has significant potential for studying chemical variations during biofilm formation.

**Figure:**
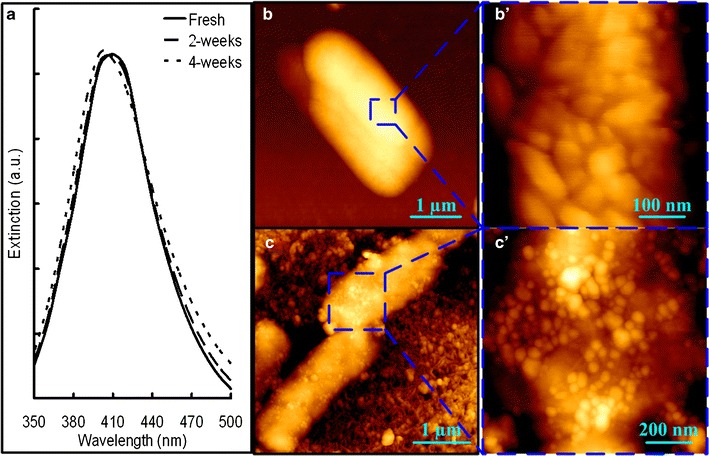
Achieving surface-enhanced Raman scattering by coating silver nanoparticles on biofilm surface.

Achieving surface-enhanced Raman scattering by coating silver nanoparticles on biofilm surface.

## Introduction

It is well documented that bacteria may form biofilms via several general phases [1], including initial reversible attachment, irreversible attachment, bacterial colony formation, mature biofilm development, and cell dispersion. During the above processes, the bacteria develop a hydrogel-like biofilm matrix containing extracellular polymeric substances (EPS) to form a complex 3-D architecture [2]. EPS are biopolymers consisting of polysaccharides, proteins, nucleic acids, lipids, as well as humic-like substances, and could account for more than 90 % of biofilm dry mass in most biofilms [3]. Since the biofilm matrix could protect the embedded cells against harmful conditions, e.g., physical shocks (desiccation or ultraviolet radiation), chemical exposure (biocides or antibiotics), and biological processes (protozoan grazers or host immune defenses) [4], fully understanding the chemical composition and variation of EPS during biofilm formation could facilitate the enhancement of biocide efficiency, development of antifouling strategies, as well as optimization of biological wastewater treatment [5].

Confocal laser scanning microscopy (CLSM) was widely used in biofilm studies [6–10]. Various probes were applied to stain cells and EPS for observing the 3-D structure of the biofilm and quantifying the EPS content on micron scales. However, since EPS are complex mixtures which contain a large number of chemicals [5], it is difficult to design a suitable protocol to stain the whole EPS, and this limits further application of CLSM in EPS identification and quantification. Other techniques such as transmission electron microscopy (TEM) [8, 11, 12] and Fourier transform infrared spectroscopy (FTIR) [13–15] were also used to characterize the chemical composition of biofilms. These studies provided detailed information about the chemical structure of biofilm EPS. However, these techniques are also limited by several disadvantages. The pretreatment procedures of TEM, including freezing and fixations, might alter the natural structure of the biofilm or create artifacts. While for FTIR, the limitation of spatial resolution (in the range of 10 μm) may block its performance on smaller samples, such as the bacterial cells or (micro-) colonies [16].

Raman microscopy (RM) is a nondestructive analytical technique which is based on the molecular vibrations derived from the interactions between photons and molecules and provides fingerprint spectra with high spatial resolution [16, 17]. The Raman spectrum contains various information about chemical composition [16] and is being widely used for single-cell, bacterial colony, or biofilm analysis [18, 19]. However, RM still faces a significant disadvantage of inefficient signal since Raman scattering is a quite rare event which involves only one in 10^6^–10^8^ of the photons scattered [20]. A longer exposure time or more powerful laser source may be required to conquer the weak scattering signal and unfortunately may bring damage to the samples, especially to biological samples [21]. Many studies were conducted applying surface-enhanced Raman scattering (SERS) since it could give an enhancement of up to 10^6^ in scattering efficiency over normal Raman scattering [20, 22]. This technique was widely used for rapid identification of different microorganisms, including yeast, bacteria, and viruses [23–28]. Ivleva et al. [5, 29] recently applied SERS to analyze the matrix of multispecies biofilm and proved SERS had great potentials for identifying biofilm matrix components and characterizing their distribution in a biofilm even at low biomass concentration. However, as far as we know, studies were rarely conducted to characterize the chemical variation in different phases during biofilm growth by SERS. Without such data, it is difficult to comprehensively understand the biofilm matrix composition and their variation during whole biofilm development processes.

To address the above research gaps, in the present study, we evaluated the chemical variations in the matrix of biofilm at different growth phases of three typical bacteria (i.e., *Escherichia coli*, *Pseudomonas putida*, and *Bacillus subtilis*) by SERS using hydroxylamine hydrochloride-reduced colloidal silver nanoparticles (AgNPs). Meanwhile, atomic force microscopy (AFM) was also applied to study morphology change during biofilm formation processes.

## Materials and methods

### Bacterial species

Two Gram-negative bacteria, *E. coli* wild-type strain K-12 and *P. putida* DSM 291 type strain, were purchased from the *E*. *coli* Genetic Stock Center (Department of Biology, Yale University) and Deutsche Sammlung von Mikroorganismen und Zellkulturen GmbH (DSMZ), respectively. The Gram-positive bacterium *B. subtilis* ATCC 6633 was purchased from Difco Laboratories (Detroit, USA).

### Substrata preparation

All the experiments were conducted on the polished crystal quartz optical windows with thickness of 2 mm and diameter of 20 mm (QPZ20-2, CRYSTRAN, UK). Before use, the windows were immersed in ethanol/HCl (*v*/*v*, 70:1) solution overnight, washed thoroughly with sterilized deionized water, and finally heated at 550 °C for 6 h in a furnace. The prepared windows were stored in the safety cabinet before use.

### AgNP preparation

**Fig. 1:**
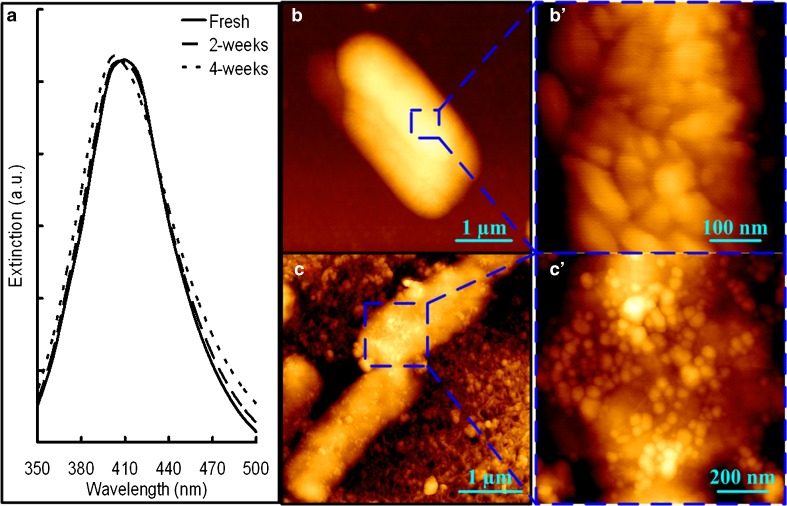
UV–vis spectra of prepared AgNPs (**a**). AFM height images showing the *E. coli* cells without (**b** and **b′**) and coated with (**c** and **c′**) the prepared AgNPs

AgNPs with diameter of 20 to 30 nm [5] were prepared by reduction of silver nitrate with hydroxylamine hydrochloride at alkaline pH and room temperature [30]. Briefly, 10 mL of silver nitrate (10^−2^ M) was rapidly added, while stirring, into 90 mL of premixed solution containing hydroxylamine hydrochloride (1.67 × 10^−3^ M) and sodium hydroxide (3.33 × 10^−3^ M). Then, the solution was kept stirring for 30 s to facilitate the completeness of the overall reaction. The UV–visible spectrum analysis indicated that the AgNPs had maximum absorption at 408 nm with a full width at half maximum of about 80 nm (Fig. 1). The AgNP solution was stored in darkness and 4 °C. The AgNPs could remain stable within 4 weeks by this means of storing (Fig. 1) [5].

### Cell cultures and preparation

The bacteria were cultivated at 150 rpm in standard Luria-Bertani (LB) medium (10 g of tryptone, 5 g of yeast extract, and 10 g of NaCl per 1 L of deionized water, pH adjusted to 7.2 and sterilized at 121 °C for 20 min). The cultivation temperature for *E. coli* and *P. putida* was 37 °C. For *B. subtilis*, the cultivation temperature was controlled at 30 °C. Cells were harvested in the stationary phase after 24 h cultivation. The bacteria cells were collected by centrifugation (3,000 rpm, 4 °C, 10 min) and washed three times in 165 mM phosphate buffer saline (PBS, 1.093 g Na_2_HPO_4_, 0.276 g NaH_2_PO_4_, and 8.475 g NaCl in 1 L deionized water, pH adjusted to 7.2 and sterilized at 121 °C for 20 min) to remove the residual LB medium. Bacterial cells were resuspended in PBS to a concentration equivalent to an optical density at 600 nm (OD_600 nm_) of about 0.2. The suspension was then used for cell adhesion and biofilm cultivation immediately.

### Cell adhesion and biofilm cultivation

First, 200 μL of prepared cell solution was added on the surface of 10 pieces of prepared quartz windows and cultivated at room temperature for 1 h. After that, the windows were carefully washed three times by 165 mM PBS solution to remove unbound cells. Then, two of the windows were conducted RM and AFM tests immediately to evaluate the bacterial initial attachment. And, the remaining eight windows were immersed in the diluted LB medium (0.1 g of tryptone, 0.05 g of yeast extract, and 10 g of NaCl per 1 L of deionized water, pH adjusted to 7.2 and sterilized at 121 °C for 20 min) in the sterile Petri dishes and incubated without shaking at room temperature for 4, 8, 24, and 72 h. After each cultivation period, two windows were gently washed three times with 165 mM PBS to remove suspended cells and residual medium. Then, one window was conducted the AFM test immediately after drying at room temperature for 1 h. For another window, 200 μL of prepared AgNP solution was added on the surface and dried under darkness before the following RM test. The cultivation procedures were repeated twice in the present study.

### AFM test

AFM images were acquired by using the tapping mode of JPK NanoWizard AFM (JPK Instruments, Germany). Silicon cantilever NSC14 (MIKROMASCH, Estonia) with a resonance frequency of 160 kHz and a spring constant of 5.7 N/m was applied to analyze the morphology of different biofilm phases in air. To decrease the applied force between the cantilever tip and bacteria/biofilm to minimize the influence to the bacterial/biofilm morphology during AFM scanning, the set point value of the oscillation amplitude was maintained higher (1.2 V or above) than the free amplitude of the cantilever (normally 1.0 V) [31]. The scan sizes were 10 × 10, 20 × 20, and 40 × 40 μm^2^ based on the different phases during biofilm formation. And, the typical scan rate in the present study was kept at 0.2 Hz, resulting in approximately 47 min for one scan. Six to eight areas were randomly selected for AFM scanning for each sample.

### RM test

All the SERS spectra were obtained by using a Renishaw inVia Raman microscope (Renishaw, UK) equipped with a He–Ne laser (633 nm, 17 mW). A Leica microscope (DM 2500 M, Leica Microsystems CMS GmbH, Germany) was coupled with the Raman spectrometer. The spectrometer was equipped with a grating of 1,800 lines/mm, and the detector was a Peltier air-cooled CCD array detector. Before measurement, the wavenumber calibration of the Raman system was conducted by using a silicon wafer as reference (520 cm^−1^) according to the previous studies [5, 17, 21, 29].

Two-hundred microliters of prepared AgNP solution was added on the attached bacteria or biofilms on quartz windows to achieve SERS prior to the measurements according to the previous studies [5, 24, 29]. The AFM test showed high coverage of AgNPs on the biofilm surface by this method (Fig. 1). Then, the samples were stored under darkness and dried at room temperature. The laser beam was focused on the individual cells or bacterial layer by applying the Leica ×100 (NA 0.85) objective to a spot of approximately 1 μm diameter. A confocal pinhole with 250 μm diameter was applied during measurement to enable a depth resolution of 2 to 3 μm. By this depth resolution, the biofilm bulk matrix with 1 to 1.5 μm thickness could be analyzed, which may contain more than five layers of bacterial cells for mature biofilms as the height of dried bacterial cells was about 0.2 μm [31]. The accumulation time for one spectrum was 10 s. Baseline correction was performed for better comparison according to a previous study [32], since high fluorescence background frequently appeared in the spectra of the present study. For initial adhered cells, single-cell spectra were acquired. However, for the other phases of biofilm, a spot on the bacterial layer was randomly selected since it was difficult to identify a single bacterial cell in a colony or mature biofilm. For each sample, SERS spectra of 20–25 single cells or spots on the biofilm were recorded (resulting in a total of 40–50 cells or spots for statistical analysis since the experiment was repeated twice).

## Results

### Biofilm formation

**Fig. 2:**
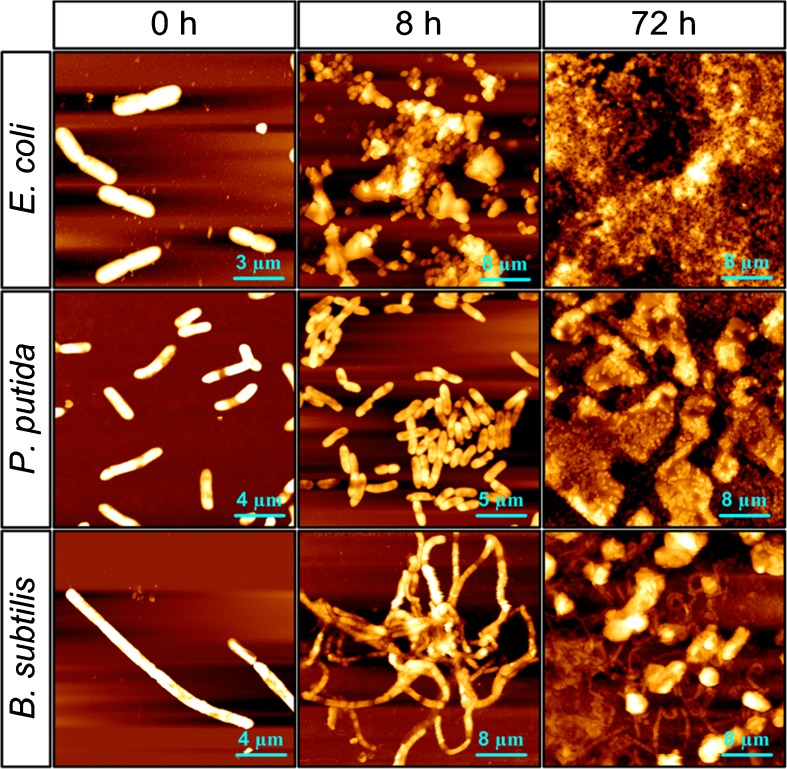
AFM height images showing the morphology of *E. coli*, *P. putida*, and *B. subtilis* biofilms after 0, 8, and 72 h cultivation

The biofilm morphology at cultivation periods of 0, 4, 8, 24, and 72 h, obtained by AFM measurement, was partially shown in Fig. 2. At 0 h, single *E. coli* and *P. putida* cells evenly adhered on the substratum, while *B. subtilis* formed multicellular chains and separately attached on the quartz surface. After 4 h cultivation in the medium, the adhered bacteria developed a microcolony with the size of several to dozens of micrometers. In this phase, only *E. coli* generated the self-produced matrix of EPS since the morphology of single cells was hard to differentiate in its microcolony. Larger colonies were formed at 8 h cultivation. More cells were produced and more complex architectures were formed in the bacterial colony at this phase, especially for *E. coli*. After keeping culturing for 24 and 72 h in LB medium, a layer of mature biofilm developed on the quartz surface and a large amount of EPS could be observed for all the three tested bacteria.

### SERS spectra of three bacterial biofilms

**Fig. 3:**
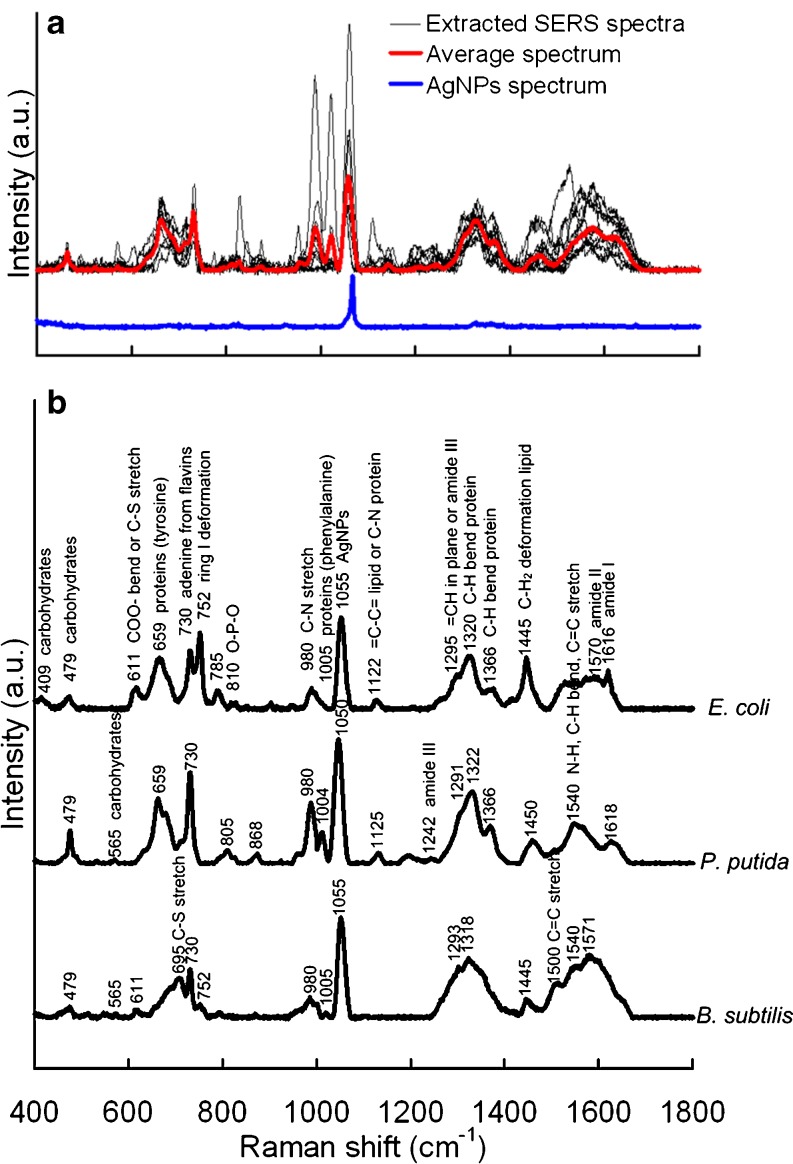
Ten extracted SERS spectra from 72 h *P. putida* biofilm and their average spectrum (**a**). The average SERS spectra (**b**) and tentative assignment of the peaks for 72 h *E. coli*, *P. putida*, and *B. subtilis* biofilms (*n* = 10)

Because of the heterogeneity of bacterial cells and different locations of the biofilm, the SERS spectra for one sample varied significantly (Fig. 3a). It was difficult to analyze the chemical variation during biofilm formation based on a single SERS spectrum. Thus, the average SERS spectrum (Fig. 3a) was applied to account for these variances and facilitate comparison between different phases of biofilm. The Raman spectrum of the applied AgNP solution was also measured and showed a high and sharp peak at around 1,055 cm^−1^, which might originate from amine bond vibrations in the prepared AgNPs. Figure 3b showed the tentative assignment of the peaks that appeared in the average SERS spectra of *E. coli*, *P. putida*, and *B. subtilis* biofilms.

These SERS spectra of biofilm contained major peaks at 479, 730, 980, 1,320, and 1,445 cm^−1^, which could be characterized as carbohydrates, nucleic acids, proteins, and lipids, according to previous studies [5, 24, 29, 33–37]. The distinct differences between the SERS spectra of Gram-negative (*E. coli* and *P. putida*) and Gram-positive (*B. subtilis*) biofilms were the peaks at 659, 1,122, and 1,366 cm^−1^, which were assigned to C–S stretching and C–C twisting proteins (tyrosine), =C–C= unsaturated fatty acids in lipids, and C–H bend proteins, respectively [33, 35, 37–39]. At other wavenumbers, such as 409, 611, 695, 752, 785, 810, 968, 1,242, 1,500, and 1,540 cm^−1^, the intensities of the peaks varied significantly, revealing that the macromolecules between biofilms were significantly different. Moreover, this might also indicate the heterogeneity of the biofilm, especially considering the limited dimension of the laser spot (*XY*, 1 μm diameter; *Z*, 2–3 μm).

### Chemical variation during biofilm formation

**Fig. 4:**
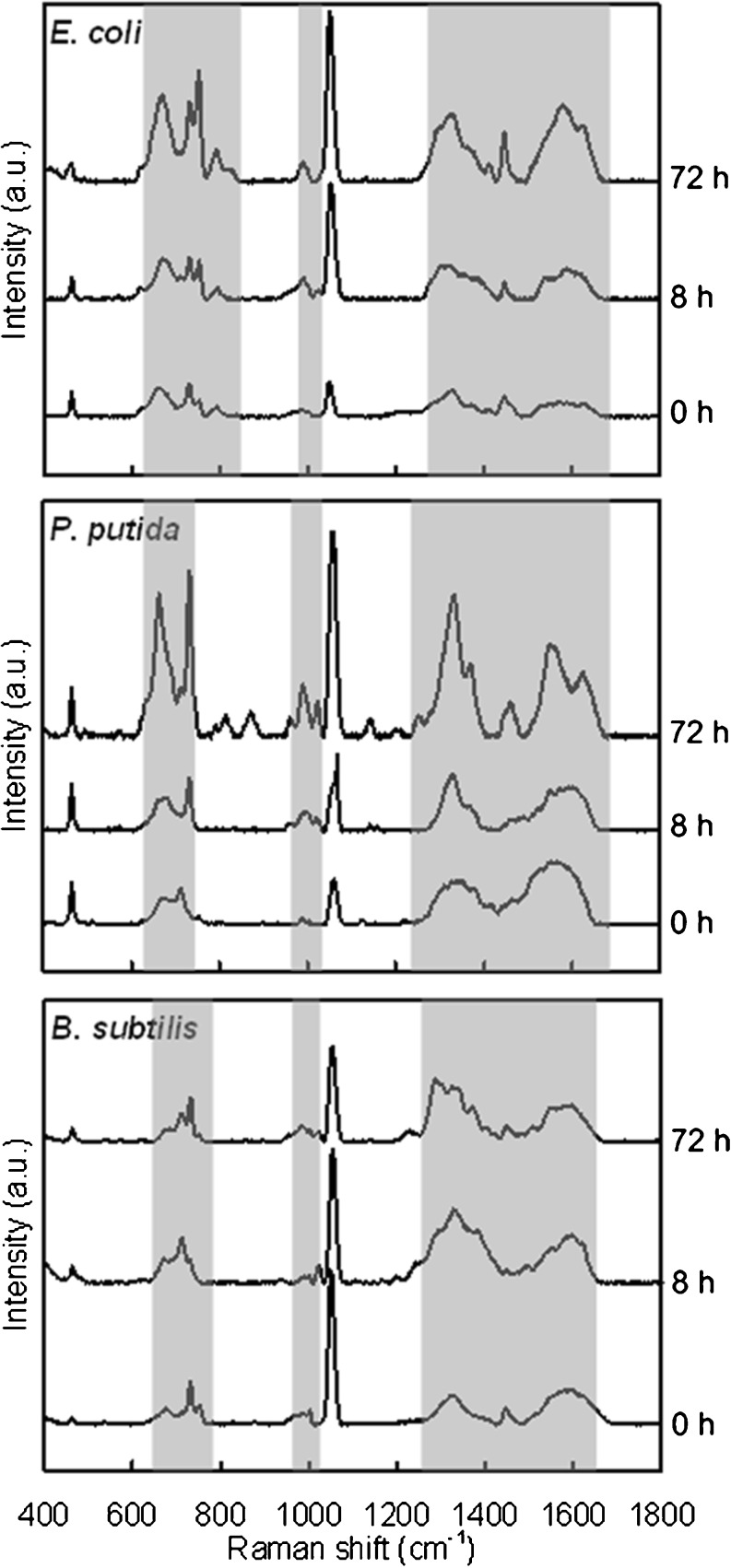
Average SERS spectra of *E. coli*, *P. putida*, and *B. subtilis* biofilms after 0, 8, and 72 h cultivation (*n* = 40–50). *Shadow regions* indicate the variation of peaks in different phases during biofilm formation

The average SERS spectra at different biofilm phases (e.g., 0 h, initial attachment; 8 h, bacterial colony; 72 h, mature biofilm) were shown in Fig. 4 to compare and observe the dynamic chemical variation during biofilm formation. The results indicated that the distinctions of different SERS spectra mainly existed in three regions, i.e., 685–800-, 950–1,010-, and 1,270–1,630-cm^−1^ ranges (shadow regions in Fig. 4).

**Fig. 5:**
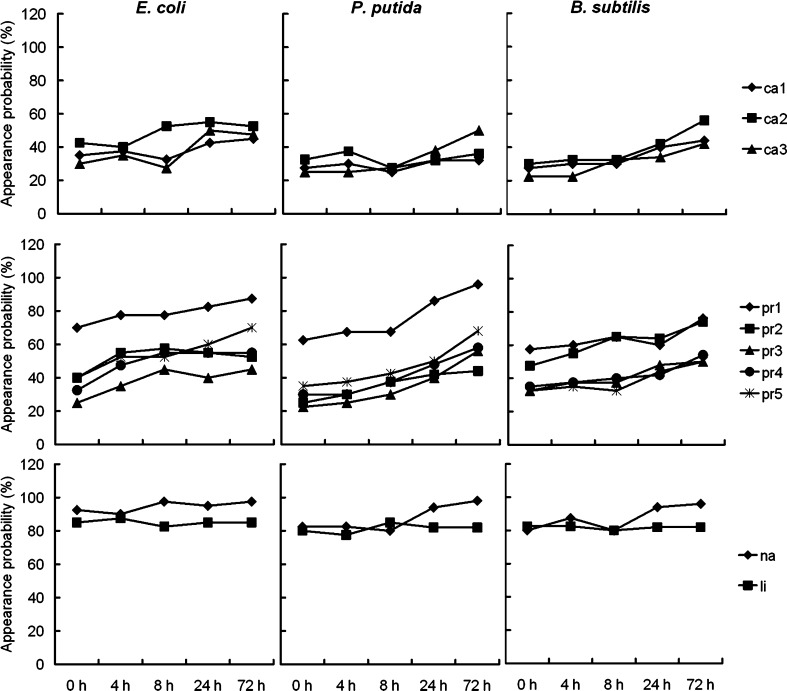
The appearance probabilities of macromolecules (carbohydrates, proteins, nucleic acids, and lipids) in the SERS spectra of *E. coli*, *P. putida*, and *B. subtilis* biofilms after 0, 4, 8, 24, and 72 h cultivation

To further study the variations of macromolecules during biofilm formation, each acquired SERS spectrum was reduced to a series of 10 peaks according to the previous study [40]. These peaks were sorted into four macromolecular classes, including three carbohydrates, five proteins, one nucleic acid, and one lipid (Table 1). The appearance probability (defined as number of peak appearance times/total number of acquired spectra × 100 %) of each peak (macromolecule) was also calculated in 40–50 SERS spectra for each sample to reflect the macromolecular content in single bacterial cells or biofilm matrix in one specific phase. Figure 5 shows the variations of macromolecules in different biofilm phases. The appearance probability of ca1 and ca3 for three bacteria increased significantly (*P*_ca1_ = 0.037 and *P*_ca3_ = 0.012) from 30 ± 4.3 and 26 ± 3.8 % at 0 h to 40 ± 6.2 and 46 ± 4.1 % at 72 h. While for ca2, the increasing trend was not significant (*P*_ca2_ = 0.188). All of these showed that the bacterial carbohydrates slightly varied during biofilm formation. For proteins, the pr1 of *E. coli* (79 ± 6.5 %) and *P. putida* (76 ± 14 %) had relative higher appearance probabilities than that of *B. subtilis* (62 ± 3.6 %). And, comparing with the 0 h (averaging 39 ± 14 %), all the proteins increased significantly (*P*_pr1_ = 0.046, *P*_pr2_ = 0.041, *P*_pr3_ = 0.041, *P*_pr4_ = 0.013, and *P*_pr5_ = 0.030) after 72 h cultivation (averaging 62 ± 15 %), indicating the protein contents varied intensively from bacterial initial attachment to mature biofilm. The appearance probability of nucleic acid also slightly increased (*P* = 0.077) from 85 ± 6.6 % at 0 h to 97 ± 1.0 % at 72 h cultivation. While for lipid, no significant variation (*P* = 0.580) was observed, and the appearance probability of lipid was maintained stably at 83 ± 2.5 %.

**Table 1:** Subset of SERS spectra peaks used for each analysis

Raman shift (cm^−1^)	Tentative assignment	Macromolecular assignment	Code	References
408–423		Carbohydrates	ca1	[35, 37]
479–495		Carbohydrates	ca2	[35, 37]
565–582		Carbohydrates	ca3	[33, 35]
637–695	C–S stretching and C–C twisting of proteins (tyrosine)	Proteins	pr1	[33, 35, 37]
727–734	Adenine from flavin	Nucleic acids	na	[29, 34, 37]
1,000–1,010	C–C aromatic ring stretching (phenylalanine)	Proteins	pr2	[5, 35–37]
1,235–1,260	Amide III	Proteins	pr3	[29, 33, 36]
1,440–1,455	C–H_2_ deformation	Lipids	li	[29, 37]
1,571, 1,572	Amide II	Proteins	pr4	[24, 38]
1,610–1,637	Amide I	Proteins	pr5	[29, 34–36]

In addition to the appearance probability, the intensities of the selected 10 peaks in different biofilm phases were also recorded and shown in Table 2. Different from appearance probability, the peak intensity of three carbohydrates for three bacteria showed no significant increasing trend (take *E. coli* as example: *P*_ca1_ = 0.076, *P*_ca2_ = 0.816, and *P*_ca3_ = 0.661) after 72 h cultivation, since large differences in intensity, mainly caused by their low appearance probabilities (Fig. 5), existed in the peaks of ca1, ca2, and ca3 in the acquired SERS spectra. For proteins, the peak intensity of pr1 and pr4 in *E. coli* largely increased 2.6 (*P*_pr1_ = 0.003)- and 4.6 (*P*_pr4_ = 0.012)-fold after 72 h cultivation, respectively. While for the other three proteins, the increased trends were not significantly observed in the present study. Similar results were also obtained in *P. putida*. However, for *B. subtilis*, no significant variation of five proteins was observed (*P*_pr1_ = 0.421, *P*_pr2_ = 0.540, *P*_pr3_ = 0.274, *P*_pr4_ = 0.776, and *P*_pr5_ = 0.914) by comparing peak intensities in the SERS spectra of 0 and 72 h. Similar with the appearance probability, the peak intensities of nucleic acid in *E. coli* and *P. putida* also significantly increased (*P*_*E. coli*_ = 0.006 and *P*_*P. putida*_ < 0.001) after 72 h cultivation. For lipid, the results were different from the appearance probability that significant increase of lipid intensity could be observed in *E. coli* (*P* = 0.030) and *P. putida* (*P* = 0.023). However, for *B. subtilis*, such augmentation cannot be significantly observed (*P* = 0.311).

**Table 2:** The peak height of 10 selected peaks in *E. coli*, *P. putida*, and *B. subtilis* biofilms after 0, 8, and 72 h cultivation (*n* = 40–50)

Code	*E. coli*			*P. putida*			*B. subtilis*		
	0 h	8 h	72 h	0 h	8 h	72 h	0 h	8 h	72 h
ca1	26 ± 21	35 ± 50	197 ± 239	26 ± 31	39 ± 52	69 ± 67	114 ± 104	195 ± 198	89 ± 114
ca2	276 ± 154	251 ± 204	267 ± 263	488 ± 173	540 ± 158	637 ± 177	147 ± 131	266 ± 200	225 ± 99
ca3	20 ± 29	75 ± 60	23 ± 44	14 ± 15	52 ± 46	87 ± 51	30 ± 33	44 ± 59	57 ± 50
pr1	384 ± 328	438 ± 310	1008 ± 415	257 ± 171	332 ± 194	1398 ± 237	213 ± 134	434 ± 267	350 ± 279
pr2	111 ± 105	153 ± 105	257 ± 221	17 ± 38	218 ± 316	439 ± 408	219 ± 136	345 ± 254	174 ± 108
pr3	68 ± 92	72 ± 80	104 ± 107	129 ± 76	94 ± 68	169 ± 228	110 ± 90	318 ± 452	285 ± 206
pr4	260 ± 219	606 ± 465	1202 ± 697	468 ± 351	641 ± 516	1099 ± 542	512 ± 455	653 ± 551	692 ± 509
pr5	282 ± 325	545 ± 522	965 ± 826	400 ± 330	508 ± 242	871 ± 526	563 ± 327	635 ± 563	528 ± 195
na	437 ± 201	495 ± 186	935 ± 476	365 ± 217	606 ± 350	1959 ± 485	708 ± 212	807 ± 489	839 ± 335
li	272 ± 105	270 ± 117	558 ± 285	174 ± 113	249 ± 184	546 ± 394	287 ± 152	206 ± 212	470 ± 431

Data are mean values ± standard deviations

## Discussion

It should be noticed that the results presented in this study showed significant variations of the extracted SERS spectra for one biofilm (Fig. 3a). The low repeatability of SERS spectra for biofilm samples might be mainly caused by the natural chemical heterogeneity of bacterial colonies and biofilms. Similar variations were also reported in previous studies by others on biofilm heterogeneity by applying different methods, including CLSM [9], FTIR [14], normal Raman [17], and SERS [29], which strongly suggested that the biofilms have a heterogeneous structure with non-uniform distribution of various chemicals. The chemical heterogeneity in biofilms can be attributed to the microscale heterogeneity in solute chemistry that is present within the biofilm matrix [41], such as the concentration gradients of oxygen, nutrients, and metabolic products. This is also consistent with the spatial dimension (*XY*, 1 μm diameter; *Z*, 2–3 μm) of the laser spot applied on the samples. To eliminate the variances in the extracted SERS spectra for one sample and facilitate the following comparison between different phases of biofilm, we collected abundant SERS spectra (*n* = 40–50) for one sample and calculated the average spectrum for peak assignment and sample comparison. In the analysis of chemical variations in different biofilm developing phases, comprehensive comparisons were also carried out in both peak intensity (Table 2) and appearance probability (Fig. 5), to obtain the reliable results.

Although many peaks that appeared in the SERS spectra in the present study could match well with those of the same bacteria in previous studies [21, 25, 26, 34, 37], several key peaks varied significantly. For example, the SERS spectra of *E. coli* single cells in the present study (Fig. 4) contained four major peaks at 659, 730, 1,320, and 1,445 cm^−1^, while other studies showed obviously different patterns or shapes of *E. coli* SERS spectra. Premasiri et al. [26] observed two most intense peaks at 732 and 1,027 cm^−1^, and Kahraman et al. [37] acquired *E. coli* SERS spectra with four major peaks, including 1,146, 1,274, 1,454, and 1,501 cm^−1^. Similar results were obtained by Kahraman et al. [21] and Culha et al. [34], in which two major peaks at ~730 and ~1,330 cm^−1^ appeared in their *E. coli* SERS spectra. For SERS spectra of *B. subtilis*, there are also differences between the present and previous studies. The two most intense peaks were at 732 and 1,330 cm^−1^ in the report of Jarvis et al. [25] and at 730 and 1,320 cm^−1^ in the present study (Fig. 4). Premasiri et al. [26] obtained more comprehensive SERS spectra of *B. subtilis* which contained three major peaks including 735, 1,080, and 1,330 cm^−1^. These large differences in SERS spectra between the previous studies and the present study might be mainly attributed to strains of tested bacteria, selected growth media, growth phases of tested bacteria (e.g., lag, growth, stationary, or death phase), wavelength of laser source, type of SERS substrate (i.e., Ag or Au), morphology of SERS substrates (e.g., colloid, rods, or surface), spatial orientation of molecular components to the SERS substrate, as well as location and coverage of the laser spot on the bacteria [26, 29, 42].

EPS are a complex mixture of biopolymers consisting of polysaccharides, proteins, nucleic acids, lipids, as well as humic-like substances [3]. Polysaccharides are a major composition of biofilm EPS and play significant functions during biofilm formation, including mediation of bacterial adhesion, formation of polymer network, protection of embedded cells, as well as retention of water and nutrients [4]. A previous study [29] proposed that the peaks at 1,555 and 1,380 cm^−1^ should predominantly appear in SERS spectra since the carboxyl groups might have direct interactions with the surface of silver colloids. However, in the present study, such two peaks were not discovered to be major peaks in three test bacterial biofilms (Figs. 3 and 4). Thus, the other three SERS peaks at 409, 479, and 565 cm^−1^, which were assigned to carbohydrates (Table 1), were applied as markers to monitor the variation of polysaccharides during biofilm formation. The results of the present work indicated that polysaccharides kept increasing significantly from bacterial initial adhesion, via (micro-) colonies, to mature biofilm (judging from appearance probabilities of the above peaks, Fig. 5). Kives et al. [43] obtained similar results when they compared polysaccharide differences between planktonic and biofilm-associated EPS in *Pseudomonas fluorescens* B52 and found that the polysaccharides in biofilm-associated EPS were much higher (two- to fourfold) than that in planktonic cells. This might be due to the polysaccharides which have been proved to be indispensable for biofilm maturation since mutants, which were unable to synthesize polysaccharides, failed to form mature biofilms [4].

Proteins, another major fraction of EPS matrix, have unique functions for biofilm development compared with other EPS compositions, e.g., enzymatic activity and electron donor or acceptor [4]. In the present study, five peaks, which were assigned to tyrosine (659 cm^−1^), phenylalanine (1,005 cm^−1^), amide I (1,616 cm^−1^), amide II (1,571 cm^−1^), and amide III (1,242 cm^−1^), were used as proteins markers (Table 1). The present study showed that the content of proteins, judging from appearance probabilities (Fig. 5) and intensities (Table 2) of the five selected peaks, increased significantly in mature biofilm after 72 h cultivation. Other previous studies obtained similar results. Ivleva et al. [17] found that the protein content in mature (>3 months) biofilm was higher than that in young biofilm by combining RM and CLSM. Kives et al. [43] also indicated that the protein concentration in a 24-h biofilm was 2.6- and 4.9-fold that in suspended cells on two individual substrata. This phenomenon could be explained by the increased density of microorganisms during biofilm formation [17].

The peak at 730 cm^−1^ in SERS spectra was considered as the well-known marker for nucleic acids [29] and was used in the present study to evaluate DNA variation during biofilm formation. The intensity (Table 2) and appearance probability (Fig. 5) of 730 cm^−1^ increased significantly from 0 to 72 h, indicating that the DNA content in the biofilm was higher than that in the bacterial colony or initial attached cells. This phenomenon might be explained by two hypotheses. First, the cell density in the mature biofilm was much higher than in a colony or on a surface in bacterial initial attachment. This might significantly increase the DNA content at the location of the laser spot. The second explanation could be the release/accumulation of extracellular DNA (eDNA) from bacterial cells into the biofilm matrix, since the eDNA, rather than intracellular DNA, could interact effectively with the added AgNPs and be largely enhanced, especially considering the short-range sensitivity of the SERS effect (normally less than 3 nm from the metal surface). eDNA was recently proved to be a major structural component in the biofilm matrix and found to play various roles for biofilm development including enhancement of adhesion [44] and cohesion of biofilm [45], as well as exchange of genetic information [4]. Palmgren and Nielsen [46] found eDNA could accumulate in the EPS matrix of activated sludge as well as pure cultures of *P. putida*. Andrews et al. [47] also found that the intensities of nucleic acids related to Raman peaks were significantly greater in biofilm cells compared with suspended cells. Moreover, Lappann et al. [48] further found that eDNA was released by genetic mediation to facilitate initial biofilm formation of *Neisseria meningitidis*. All of these indicate that, due to DNA accumulation or release, higher concentrations of eDNA might exist in the mature biofilm matrix than in earlier phases of biofilm (i.e., initial attached cells and bacterial colonies), as we observed in the present study.

Lipids were also found in the biofilm matrix and could be identified according to peaks at a range of 1,122–1,130 and 1,440–1,455 cm^−1^ [29, 47]. These peaks were also observed in our SERS spectra (Figs. 3 and 4). And, the peak at 1,440–1,455 cm^−1^ was applied to evaluate the lipid content since its intensity was higher than the peak at 1,122–1,130 cm^−1^ (Fig. 3). Although the appearance probability of lipids was kept stable, the peak's intensity increased significantly for *E. coli* and *P. putida* from bacterial initial attachment to mature biofilm (Table 2), indicating that the lipid content in the mature biofilm was higher than that in initial attached cells or bacterial colonies. However, for *B. subtilis*, such augmentation cannot be significantly observed. This might be caused by the lipopolysaccharides which only existed in the outer membrane of Gram-negative bacteria [49] and were one of the major extracellular lipids in the biofilm matrix [4]. Carotenoids were also often found in the matrix of a multispecies biofilm according to the peaks at 1,155 and 1,510 cm^−1^ [29, 47]. However, in our SERS spectra, no carotenoids were found in the biofilm of *E. coli*, *P. putida*, and *B. subtilis*, since carotenoids were proved to be typical components only for colored bacteria, such as *Rhodococcus* or *Sphingomonas* strains [47]. Two broad accompanying peaks around 1,350 and 1,600 cm^−1^ could be clearly observed in the SERS spectra (Figs. 3 and 4). These two peaks are usually ascribed to amorphous carbon which might arise from the decomposition of hydrocarbons [50] and can be assigned to humic-like substances in the biofilm according to the previous studies [17, 51]. The results indicated that the humic-like substances kept increasing along with the biofilm cultivation (Fig. 4), especially for *E. coli* and *P. putida*, demonstrating that humic-like substances, which are usually generated by degradation of organic matter, could accumulate to higher amount in mature biofilms.

The present study, as far as we are concerned, is the first detailed work which evaluated the chemical variations in different phases of biofilm growth based on the SERS technique. The chemical components in the matrix of biofilm at different phases were identified by the marker peaks in SERS spectra, and the dynamic variations of macromolecules along with biofilm growth were also analyzed. The results can be applied for the analysis of various chemical components in the biofilm matrix of a specific growth phase (such as initial attached bacteria, bacterial colonies, or mature biofilm) and may provide new information related to the relationships between structures and functions during biofilm formation. Moreover, the present study also illuminates the significant potentials for the application of SERS to characterize the complex microbiological systems, e.g., bacterial colonies and biofilm matrix. However, more SERS studies on biofilm are expected to be done in the future (1) to develop a more reproducible standardized methodology for better evaluation of the effects of different experimental factors during biofilm formation and (2) to establish a comprehensive database of the SERS spectra for microbiological samples to benefit precise peak assignment for acquired SERS spectra.
